# Supplementary material for: New science of climate change impacts on agriculture implies higher social cost of carbon
Source: Nat Commun. 2017 Nov 20;8:1607. doi: 10.1038/s41467-017-01792-x (PMC5694765; doi:10.1038/s41467-017-01792-x)
Supplement: Supplementary file 1 — Supplementary Information [file 41467_2017_1792_MOESM1_ESM.pdf]

Variable	Coefficient	Standard Error	P-Value
f(CO_2 Concentration)*C_3	17.20	5.87	0.005
f(CO_2 Concentration)*C_4	10.82	3.28	<0.001
Adaptation * ΔT	0.17	2.09	0.94
Rainfall	0.21	0.11	0.06

Supplementary Table 1: **Meta-analysis coefficients estimated from Equation 1 (Methods).** Standard errors are estimated from 1500 block bootstraps, blocking at the study level to allow for correlation between point-estimates from the same study. Significance levels are based on two-tailed hypothesis tests.

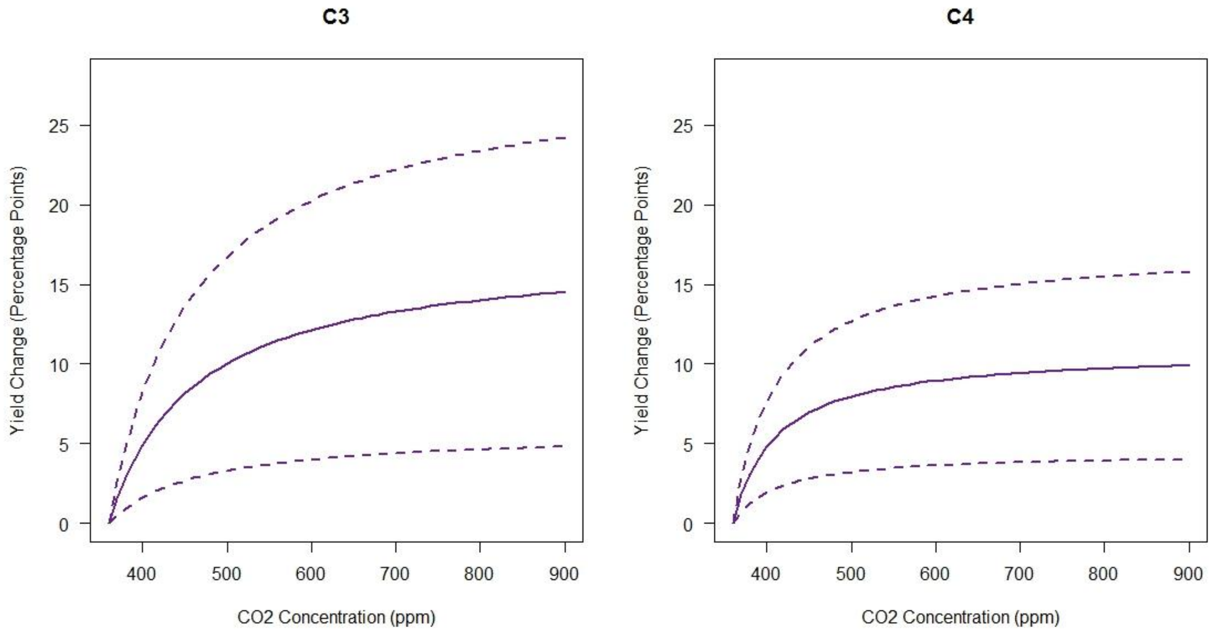

Supplementary Figure 1: Effect of CO_2 onC_3 and C_4 crop yields. Based on assumed functional form and the estimated coefficients (Table S1). A doubling of CO_2 from pre-industrial levels gives a benefit of 11.5%. for C_3 plants and 8.7% for C_4 plants.

	On-Farm, Within-Crop Agronomic Adaptations to Climate Changes			On-Farm Adaptations to Price and Productivity Changes		Changes in the Extensive Margin	Off-Farm Economic Adaptations to Price and Productivity Changes	
	Planting Date	Cultivar Choice	Input Adjustment	Crop-Switching	Production Intensification	Movement of Growing Areas	Consumption Switching	Trade Adjustment
Modeling Stage :	Meta-Analysis	Meta-Analysis	Meta-Analysis	GTAP	GTAP	GTAP	GTAP	GTAP
Notes:	(1)	(2)						

Supplementary Table 2: Description of adaptations included in the damage function and the modeling stage at which they are captured. Adaptations are defined as behavioral or management changes that reduce the negative (or increase the positive) welfare impacts of a change in climate included in the damage function. Within-crop agronomic changes that moderate the effect of climate change on productivity are captured in the adaptation term of the meta-analysis (β_8 ,Equation 1). Other adaptations that moderate the effect of productivity changes on welfare are included in the economic modeling using GTAP. Note that although adaptive behaviors by farmers have been documented in real-world settings[5–7], consistent with some of the adaptations represented in process-based crop models, no empirical validation of the magnitude of these benefits has been undertaken.

Notes: (1) 54% of studies including adaptation model changing planting date or changing planting date and cultivar; (2) 56% of studies including adaptation model changing cultivar or changing planting date and cultivar.

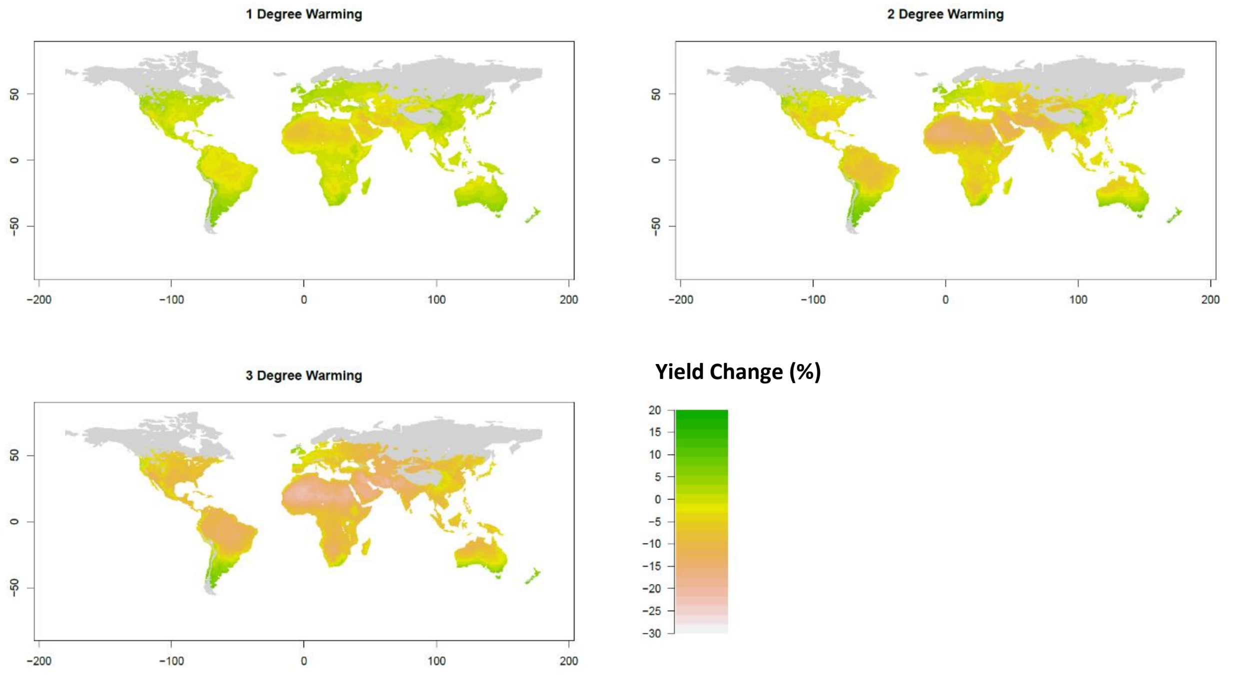

Supplementary Figure 2: Effects of climate change on maize yields. Temperate and tropical areas for 1-3 degrees of global warming. Includes adaptation and CO_2 fertilization.

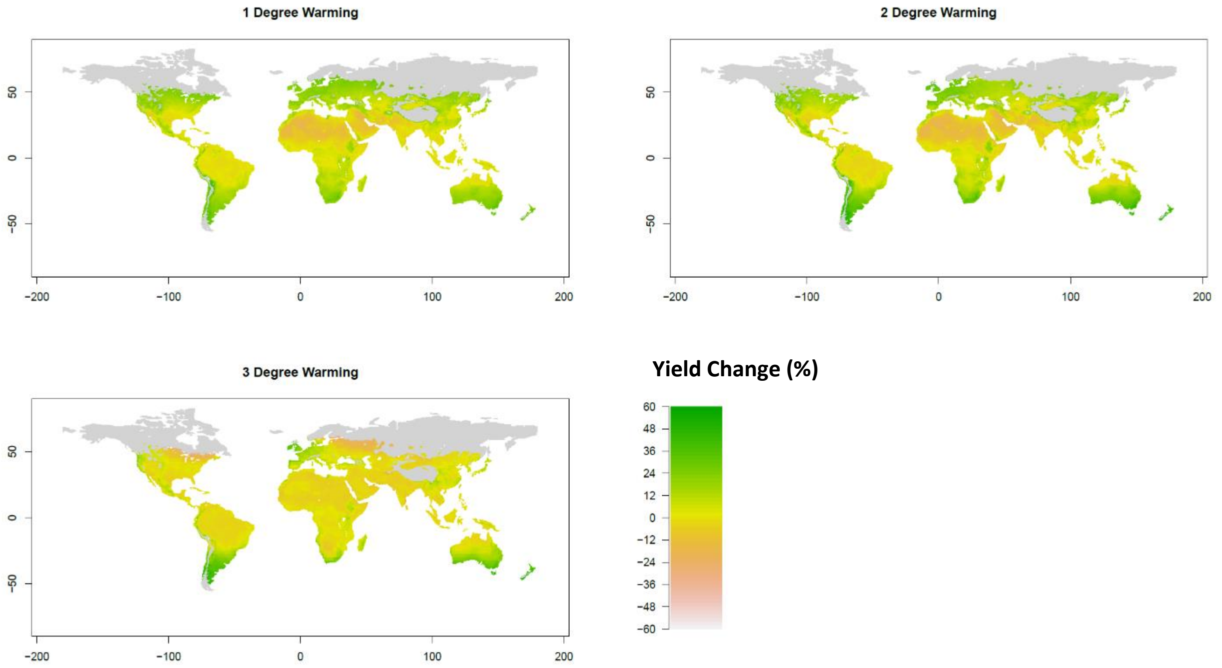

Supplementary Figure 3: Effects of climate change on rice yields. Temperate and tropical areas for 1-3 degrees of global warming. Includes adaptation and CO_2 fertilization.

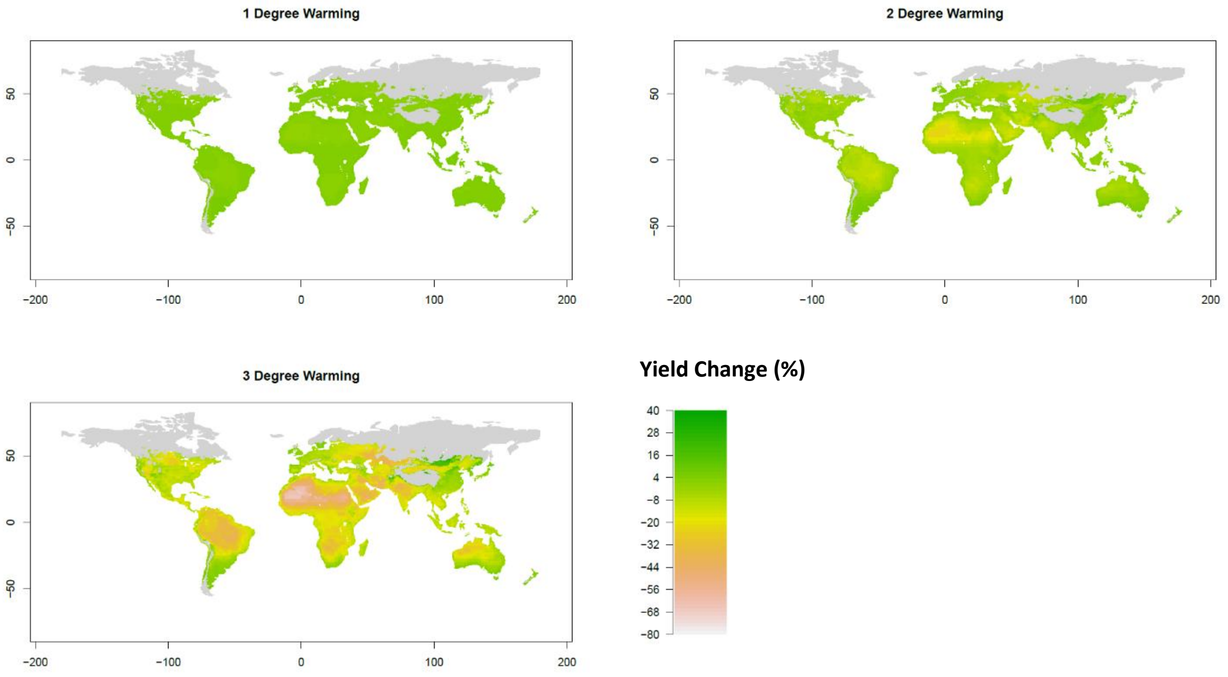

Supplementary Figure 4: Effects of climate change on wheat yields. Temperate and tropical areas for 1-3 degrees of global warming. Includes adaptation and CO_2 fertilization.

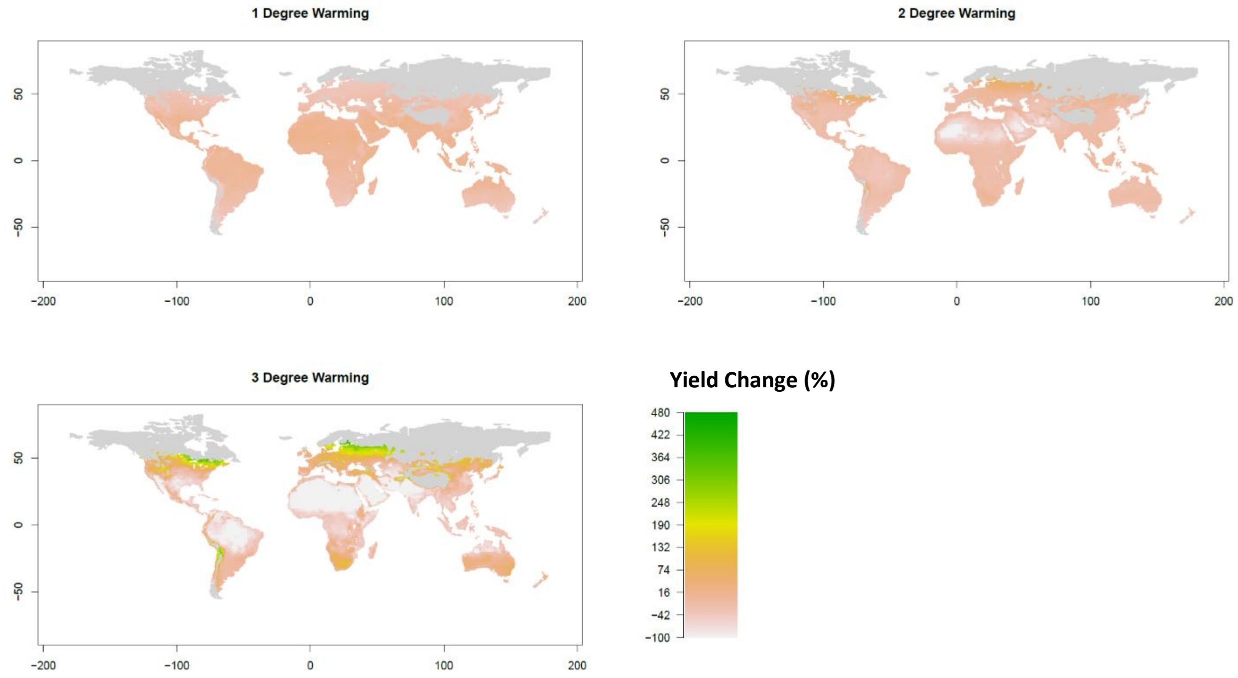

Supplementary Figure 5: Effects of climate change on soy yields. Temperate and tropical areas for 1-3 degrees of global warming. Includes adaptation and CO_2 fertilization.

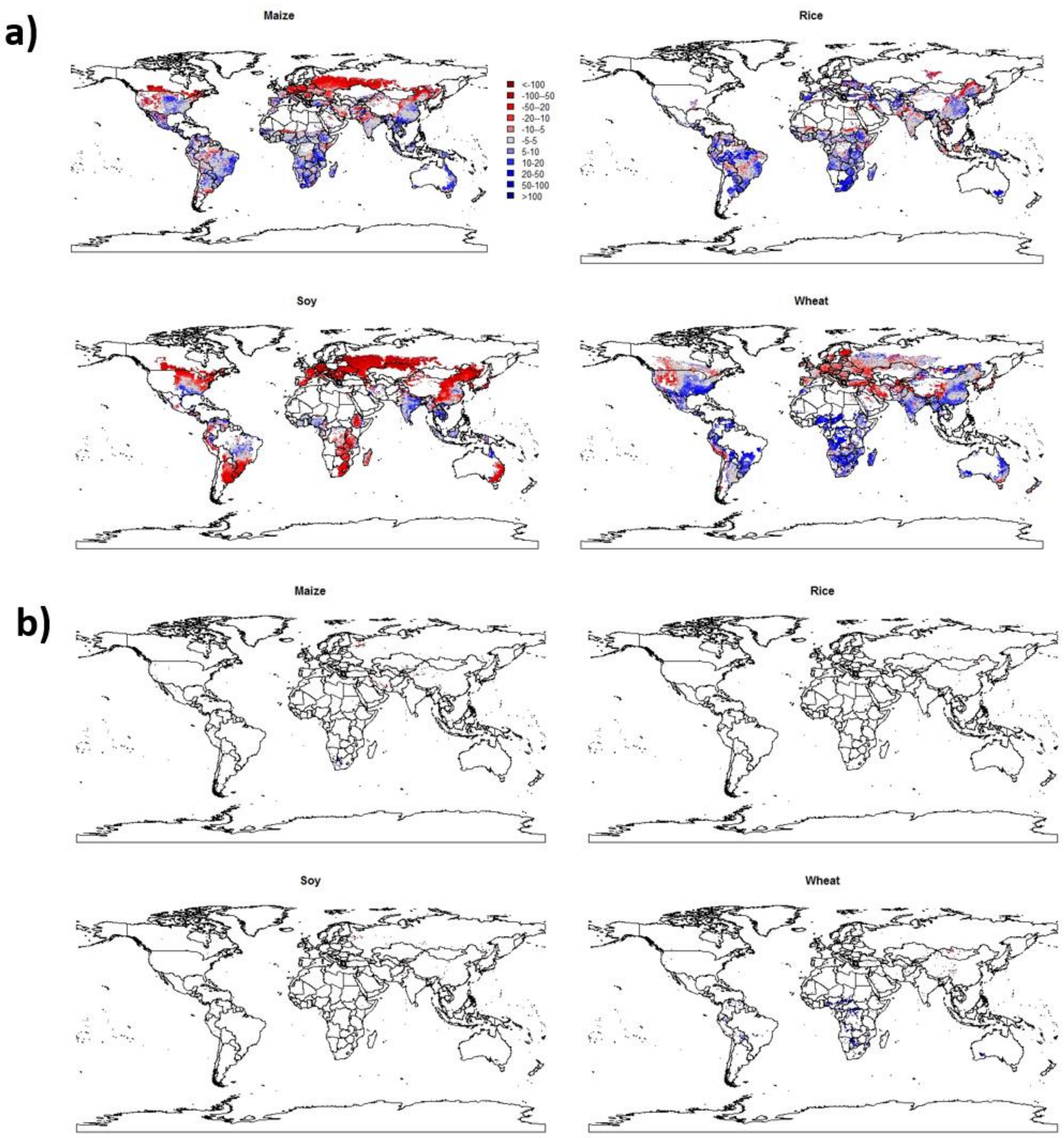

Supplementary Figure 6: Comparison between the results of the meta-analysis and the AgMIP GGCMI. Preferred ensemble using only models that explicitly represent nitrogen stress for 2°C average global warming for current crop growing areas. a) Difference in percentage points (pp) between the results of the meta-analysis presented in this paper and the AgMIP ensemble average. Grey indicates areas where the methods agree to within 5pp. Blue indicates areas where results of the meta-analysis are more optimistic than AgMIP and red areas where meta-analysis results are negative compared to AgMIP. b) Areas where there is no overlap between confidence intervals from the meta-analysis and the range of AgMIP results. Red indicates areas where the 97.5th quantile of the distribution of results from the meta-analysis results is lower than the lowest member of the AgMIP ensemble. Blue indicates areas where the 2.5th quantile of the distribution of results from the meta-analysis results is higher than the highest member of the AgMIP ensemble. (Very few areas are colored in b because the uncertainty ranges overlap almost everywhere).

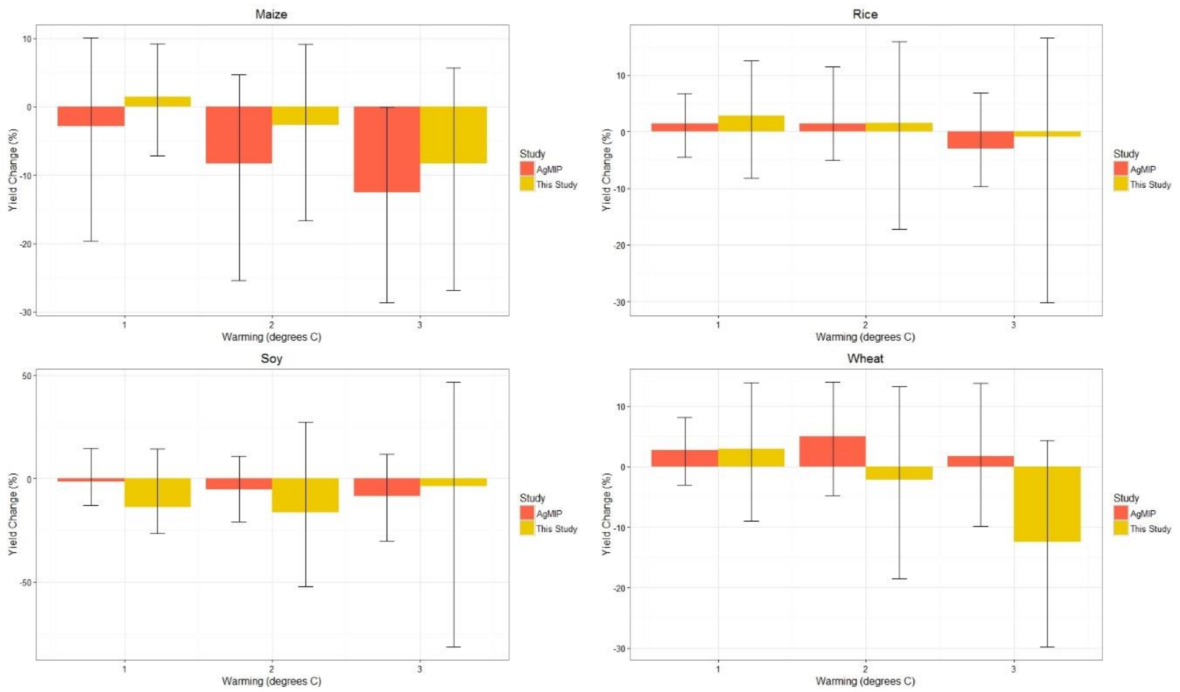

Supplementary Figure 7: Global, production-weighted change in crop yields. For global average warming of 1-3°C for the meta-analysis results presented in this study and the AgMIP GGCMI ensemble results (preferred ensemble using only models that explicitly represent nitrogen stress). Error bars show 95% range of the AgMIP distribution (AgMIP) and the 95% confidence interval for meta-analysis results (This Study) based on a block-bootstrap (Methods). Crop production areas are from 2000 and are based on Monfreda et al.[1] Results include CO_2 fertilization, adaptation (for the meta-analysis results), and irrigation in irrigated areas (AgMIP, based on Montreda et al.[1]).

**Yield Impacts**		**Welfare Impacts**	**Social Cost of Carbon**
Meta-Analysis	*AgMIP GGCMI*		
Baseline growing-season temperature	Baseline growing-season temperature	Mix of crops grown in each country and supply response	Rates of economic growth
Scaling of local to global warming	Scaling of local to global warming	Bilateral trade pattern and net trade position w.r.t each crop	Rates of population growth
	Irrigation management	Distortions (taxes and subsidies) in the agricultural sector	Size of the agricultural sector
	Soil type / quality	Consumption preferences	
	Local precipitation changes with global temperature change		
	Nutrient management (models in preferred ensemble only)		

Supplementary Table 3: Sources of spatial heterogeneity arising at each stage of the analysis. The meta-analysis averages over some sources of variation that might affect the yield response to warming and estimates a common

effect that differs by crop, baseline growing-season temperature and the relationship between local and global warming. The AgMIP GGCMI ensemble captures more sources of spatial variation. Additional spatial heterogeneity is introduced in modeling the implications of yield changes for economic welfare, which depends on the structure of the agricultural sector in each region, captured in the GTAP model. Finally, economic and population growth in the scenarios used in calculating the SCC differs by region, introducing additional spatial variation into the SCC calculation.

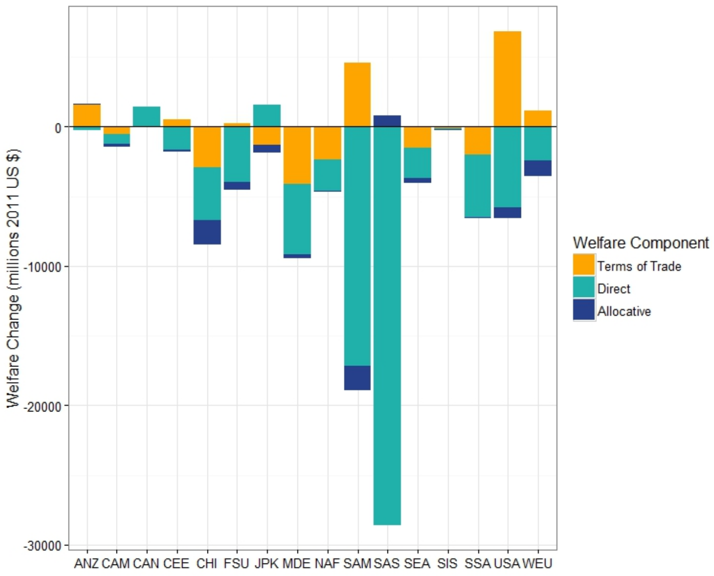

Supplementary Figure 8: Breakdown of total welfare changes for 3°C of warming. Using yield response from the meta-analysis reported in this study. Results correspond to those shown in Figure 2 but are unnormalized and aggregated to the 16 geographic regions used in the FUND model. Region definitions are given in Table S3.

FUND Region	Definition
ANZ	Australia and New Zealand
CAM	Central America
CAN	Canada
CEE	Central and Eastern Europe
CHI	China, Hong Kong, Macau, North Korea, Mongolia
FSU	Former Soviet Union
JPK	Japan and South Korea
MDE	Middle East
NAF	North Africa
SAM	South America
SAS	South Asia
SEA	South-East Asia
SIS	Small Island States
SSA	Sub-Saharan Africa
WEU	Western Europe
USA	United States of America

Supplementary Table 4: Abbreviations and definitions of the 16 geographic regions used in the FUND model.[2]

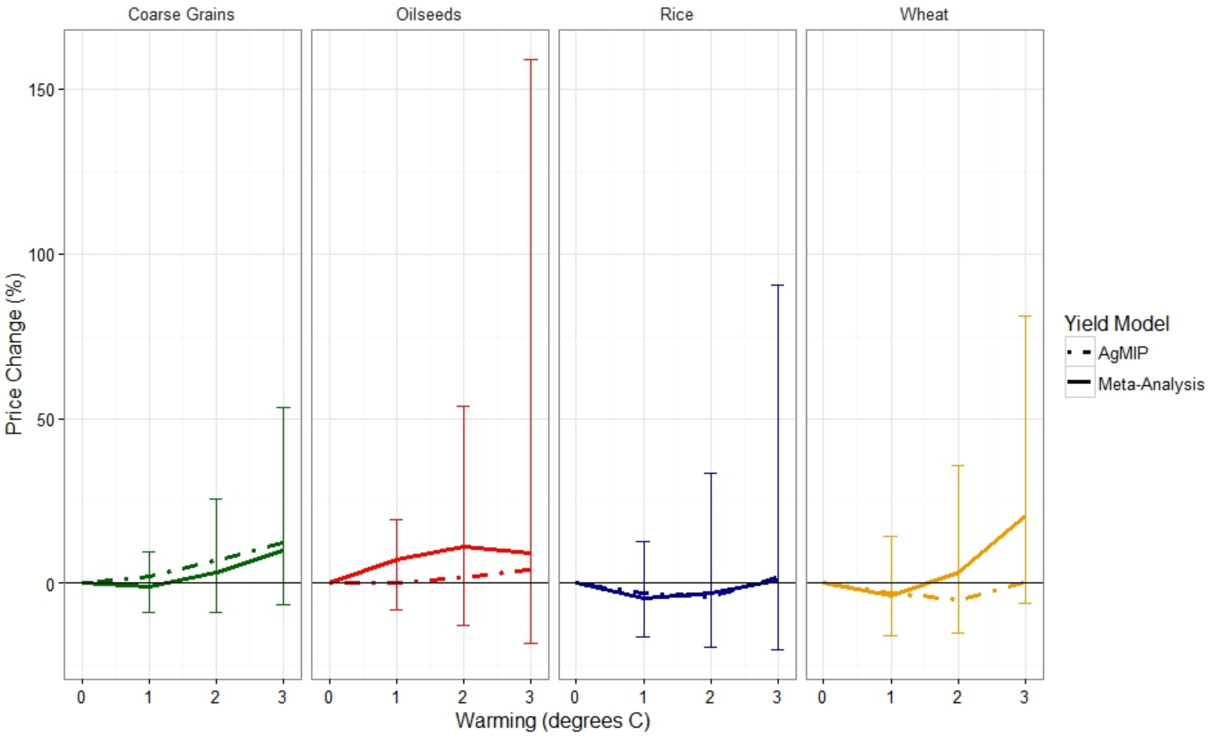

Supplementary Figure 9: World price changes within the four agricultural sectors with yield shocks examined in this study. Note that only the maize and soybeans component of the Course Grains and Oilseed sectors are shocked, not the entirety of the sector. Error bars for meta-analysis results show GTAP results based on the 2.5th and 97.5th quantile of the bootstrapped distribution of yield changes. AgMIP results are based on the GGCMI ensemble average (preferred ensemble excluding models that do not explicitly represent nitrogen stress).

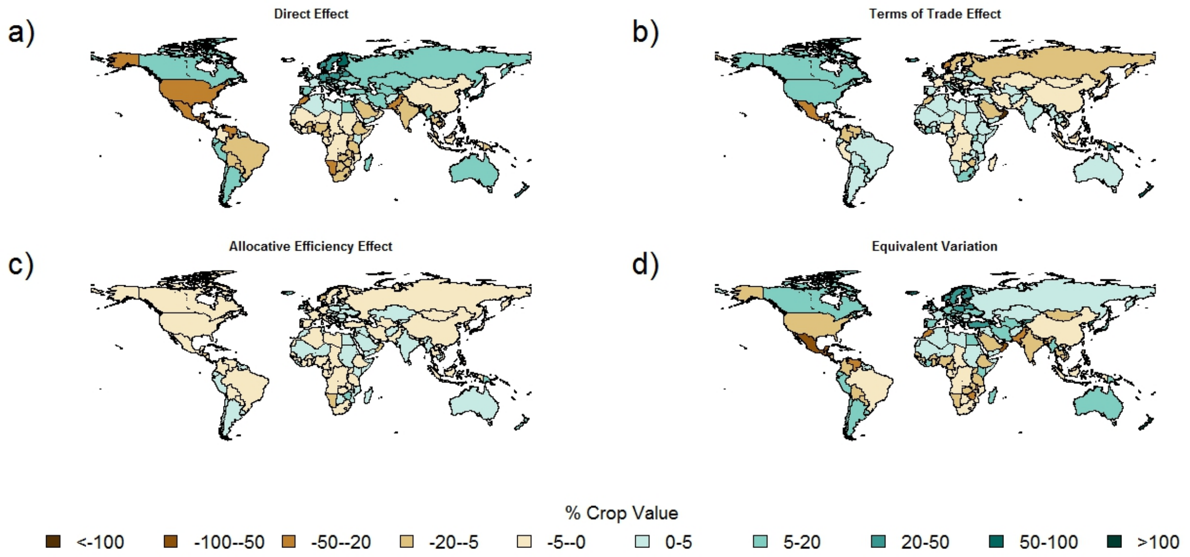

Supplementary Figure 10: Welfare changes from 3°C of global average warming. Using yield changes based on the AgMIP GGCMI ensemble mean (preferred ensemble using only models that explicitly represent nitrogen stress): a) the direct technical effect of climate change on agricultural productivity; b) terms of trade effects; c) the allocative efficiency effect and; d) total welfare change reported as equivalent variation. Results are based on yield changes that include CO_2 fertilization for all crops. Welfare changes are normalized by the value of production of the affected crops (maize, rice, wheat and soybeans).

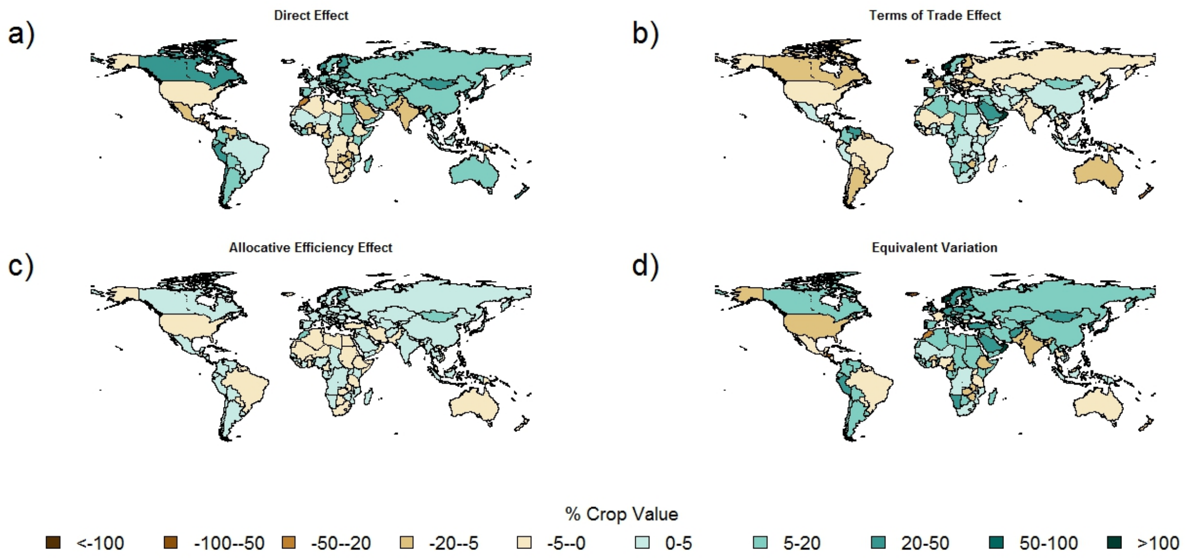

Supplementary Figure 11: Welfare changes from 3°C of global average warming. Using yield changes based on the AgMIP GGCMI ensemble mean (full ensemble): a) the direct technical effect of climate change on agricultural productivity; b) terms of trade effects; c) the allocative efficiency effect and; d) total welfare change reported as equivalent variation. Results are based on yield changes that include CO_2 fertilization for all crops. Welfare changes are normalized by the value of production of the affected crops (maize, rice, wheat and soybeans).

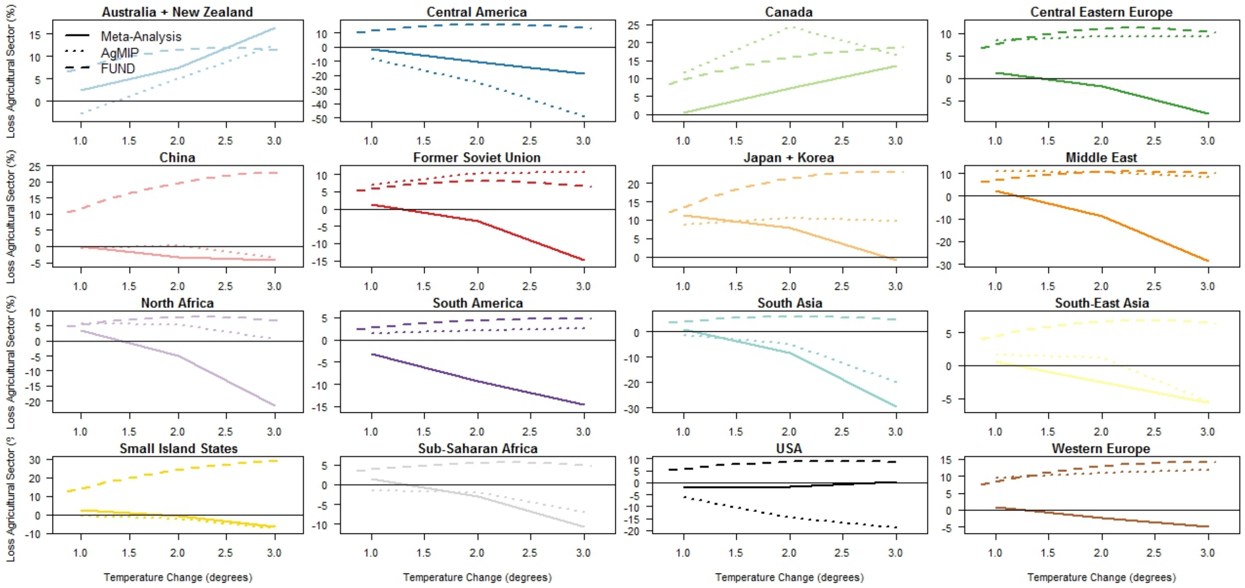

Supplementary Figure 12: As Figure 3 in main text, but excluding error bars. To enable a clearer comparison between point estimates of the different damage functions.

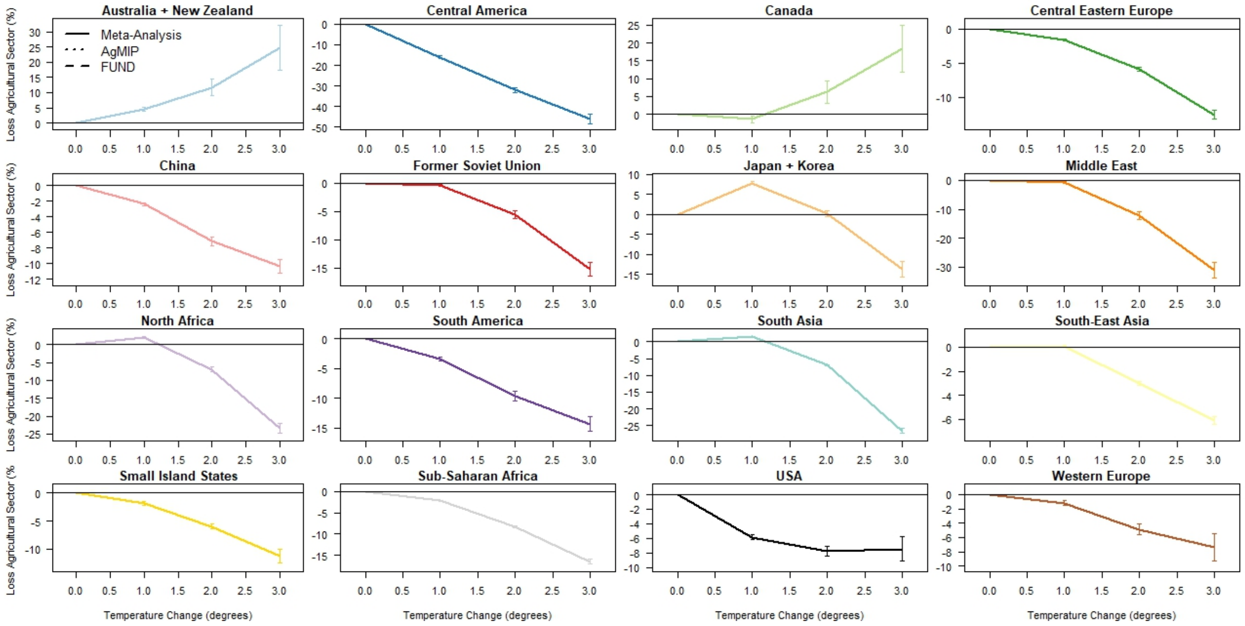

Supplementary Figure 13: Results of the GTAP sensitivity analysis. Using a 16-region version of GTAP (matching the FUND regions). Additional details in Methods and Table S7. Error bars show +/- 2 standard deviations based on a sensitivity analysis of key parameters in GTAP. Because these damage functions come from a more aggregated version of GTAP, they are slightly different from those shows in Figure 3 and Figure S12 that come from the full 140-region version of the model.

		FUND	AgMIP (All)	AgMIP (Preferred)	Meta-Analysis
Linear Interpolation	2.50%	12.25	12.57	19.20	25.11
	3.00%	8.57	9.57	14.80	19.73
	5.00%	2.97	4.22	6.76	9.55
Quadratic Fit	2.50%	12.25	15.20	21.82	27.05
	3.00%	8.57	11.05	16.39	20.79
	5.00%	2.97	4.28	6.99	9.27

Supplementary Table 5: Results of SCC sensitivity analyses. Includes yield impacts modelling, discount rate, and the interpolation of the damage function between and beyond points obtained from GTAP.

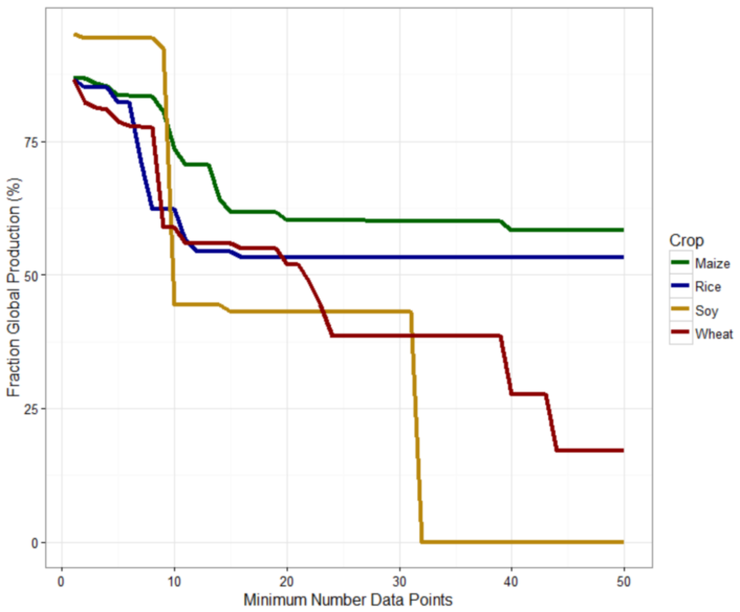

Supplementary Figure 14: Geographic representation of production in the data-base of studies included in the meta-analysis. Each study is assigned to a country (Methods) and the fraction of 2000 global production from that country used to determine the fraction of global production represented for each crop.

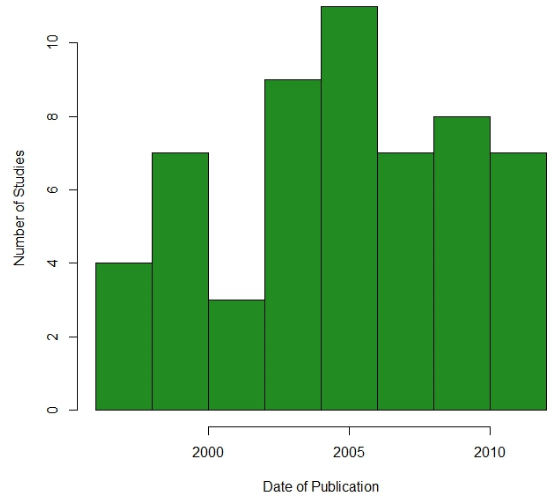

Supplementary Figure 15: The 56 studies included in the analysis by date of publication.

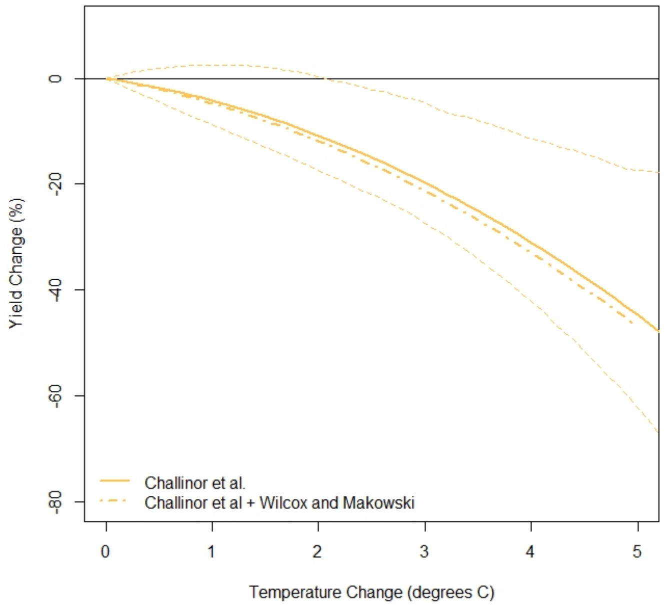

Supplementary Figure 16: Comparison of the wheat yield response curve with and without additional data-points from Wilcox and Makowski. For the median baseline temperature estimated using Equation 1 based on the database in Challinor et al[3] and based on an expanded database that adds 6 studies included in Wilcox and Makowski[4] but not Challinor et al[3]. This is the subset of observations that include data for all variables necessary to estimate Equation 1 (change in temperature, rainfall, CO_2, and whether or not the study included adaptation (limited to adaptation of sowing date in this study)). The observations from the Wilcox and Makowski database are combined with baseline growing-season temperature for growing areas in the relevant country as described in the

Methods section for the Challinor et al. database. These additions increase the number of point estimates of wheat yield from 336 to 642 and the total number of point estimates from 1010 to 1316, but do not substantively change the estimated response function. The confidence interval is the 95% interval based on the block bootstrap of the regression using just the Challinor et al database.

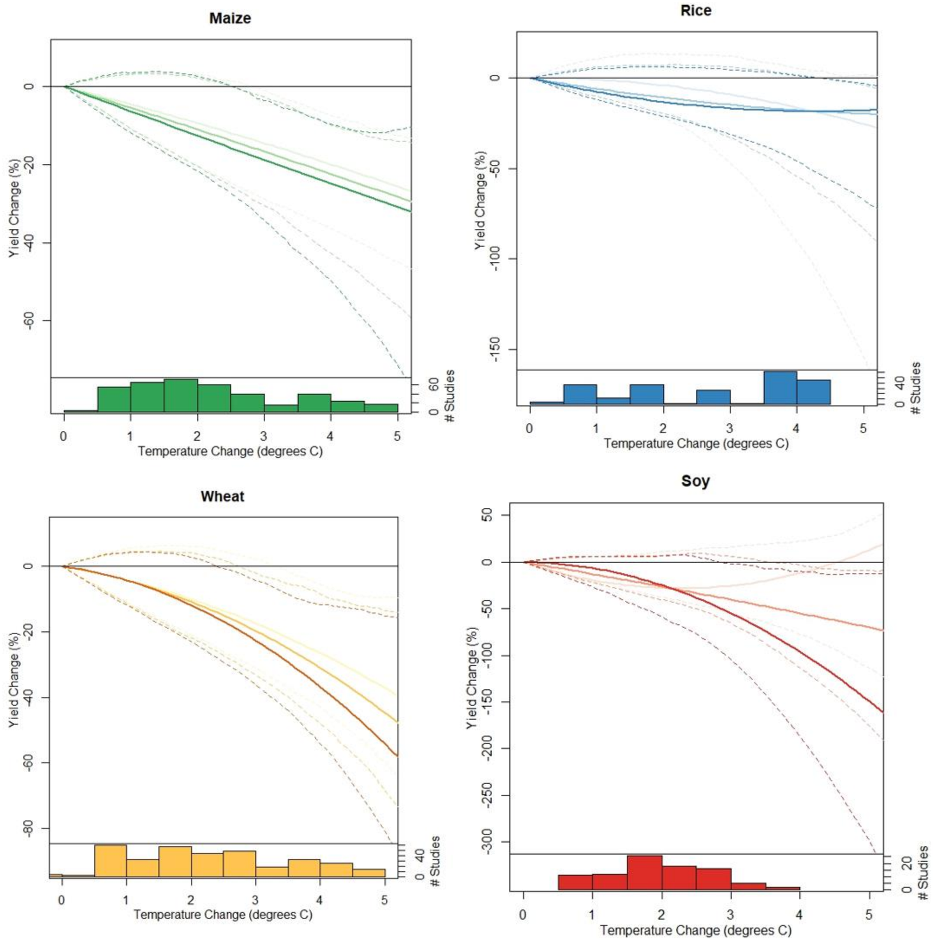

Supplementary Figure 17: As Figure 1 except with 95% confidence interval estimated using a block bootstrap blocking at the model level. Error bars are qualitatively similar to those in Figure 1, particularly for warming less than 3°C. Some crops in some areas (notably rice in cooler areas and maize in warmer areas) do show a substantially more negative lower bounds, particularly at higher levels of warming.

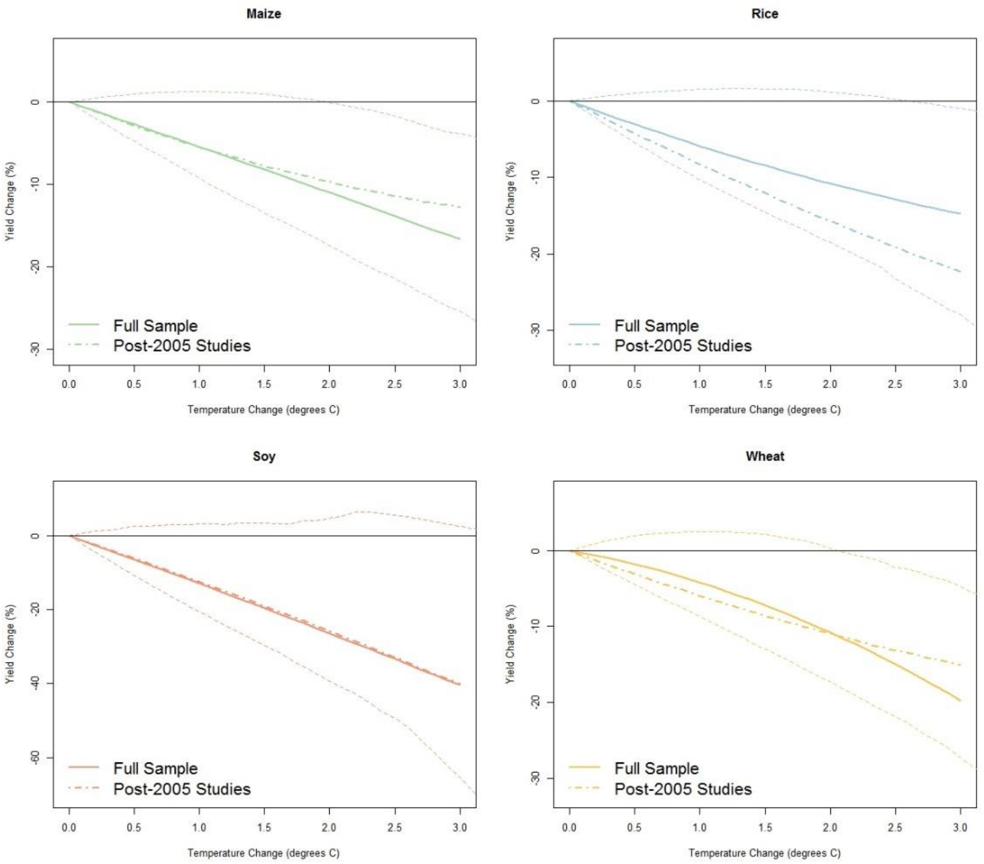

Supplementary Figure 18: Yield temperature response functions for the full data-base and for a subset of the studies published 2005 or later. Both are shown at the median growing-season temperature and do not include CO_2 fertilization or adaptation.

	Intercept Term	"True" Adaptation Term
**All Adaptations (Preferred Specification)**	6.30%	0.17% per degree C
	(4.81)	(2.09)
**Changing Planting Date**	3.06%	-4.41% per degree C
	(8.15)	(2.79)
**Changing Cultivar**	13.26%	1.23% per degree C
	(11.11)	(3.04)

Supplementary Table 6: Adaptation coefficients for the preferred model and an alternative model that includes the effect of changing planting date and changing cultivar separately. The effect of other adaptations could not be separately identified because they are not adequately represented in the yield impacts database. No coefficients are statistically different from zero at the 95% level.

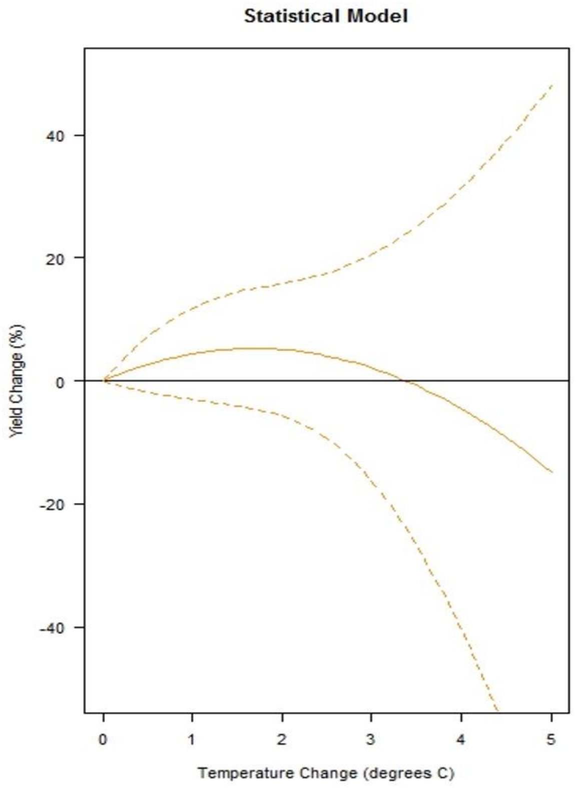

Supplementary Figure 19: The effect of type of study on temperature response. This was identified by introducing two additional terms into our preferred specification, Equation 1: $\Delta Y_{ijk} \;\; = \beta_{1j}\Delta T_{ijk} * Crop_j + \beta_{2j}\Delta T_{ijk}{}^2 * Crop_j + \;\; \beta_{3j}\Delta T_{ijk} * Crop_j * \bar{T}_{jk} + \beta_{4j}\Delta T_{ijk}{}^2 * Crop_j * \bar{T}_{jk} + \beta_5 f_1(\Delta CO_{2ijk}) * C_3 + \beta_6 f_2(\Delta CO_{2ijk}) * C_4 + \beta_7\Delta P_{ijk} + \beta_8\Delta T_{ijk} * Adapt_{ijk} + \beta_9 Adapt_{ijk} + \boldsymbol{\beta_{10}\Delta T_{ijk} * Stat_{ijk}} + \boldsymbol{\beta_{11}\Delta T_{ijk}{}^2 * Stat_{ijk}} + \varepsilon_{ijk}$. The graph shows the quadratic given by the β_{10} and β_{11} is shown in the figure, along with 95% confidence intervals from a non-parametric block-bootstrap, blocking at the study level. This can be interpreted as the difference in temperature response, controlling for other relevant study factors, between empirical and process-based studies. Error bars are very large at higher levels of warming because there are few statistical studies in the sample and they tend to report impacts at 1°C of warming. The finding that process-based and empirical studies give similar results after accounting for differences in treatment of CO_2-fertilization is consistent with findings in other recent studies[8,9].

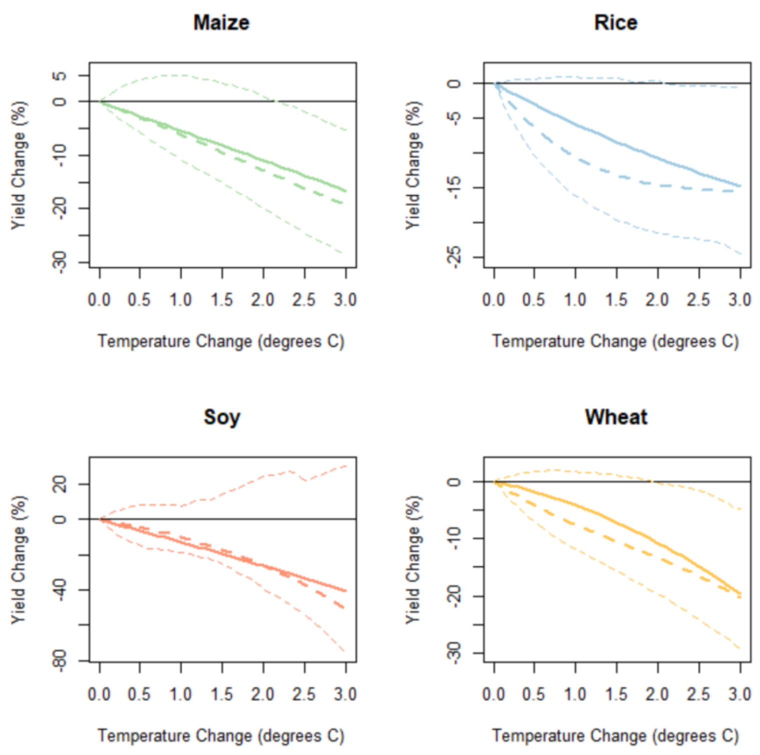

Supplementary Figure 20: Comparison of temperature response curves with and without a cubic warming term. Solid lines show the quadratic response estimated using Equation 1. Dashed lines show response with the addition of a cubic warming term. Curves are shown for the median baseline temperature. Confidence intervals give the 95% confidence intervals of the quadratic response.

Terms Removed from Regression	F-Statistic	Probability Restricted Model = Unrestricted Model
1. All crop interaction terms	11.24	<2e-16
2. All quadratic warming terms	6.083	1.2e-7
3. All interaction terms between warming and baseline temperature	14.67	<2e-16
4.1 All CO2 fertilization terms	22.47	2.9e-10
4.2 CO2 fertilization just for C4	37.57	1.27e-9
5. Rainfall Control	10.86	0.001
6. All adaptation terms	2.57	0.07

Supplementary Table 7: F-Tests of the effect of removing individual terms from the Equation 1. There is strong evidence that all terms add explanatory power to the model, with the slight exception of the adaptation terms. These terms nevertheless have to be included because economic theory requires that climate damage functions account for the benefits of adaptation.

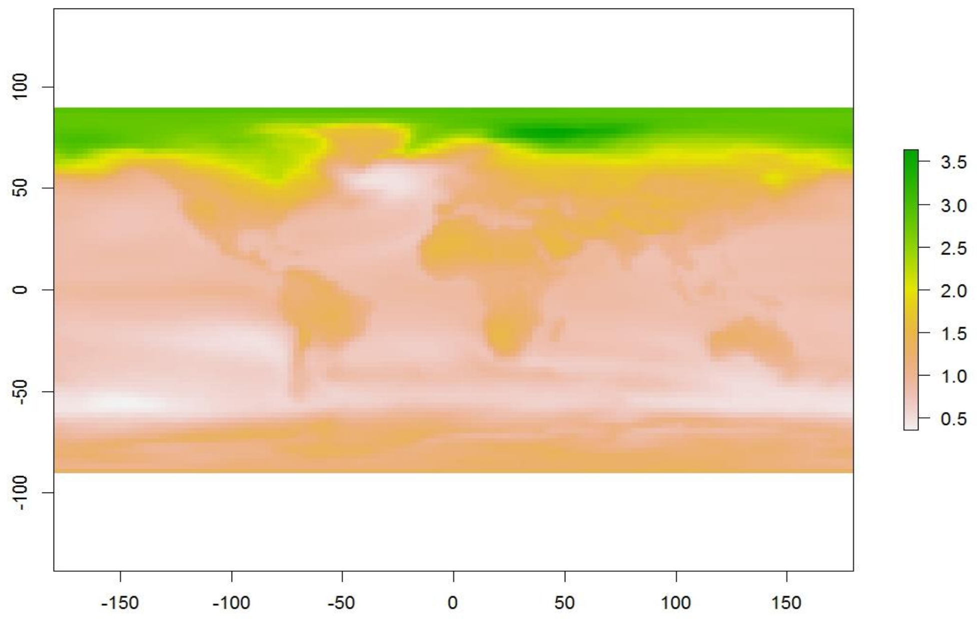

Supplementary Figure 21: Local change in temperature per degree increase in average global temperature. Based on the CMIP5 multi-model ensemble mean RCP 8.5 scenario using temperature changes between and end period of 2035-2065 and a baseline period of 1861-1900.

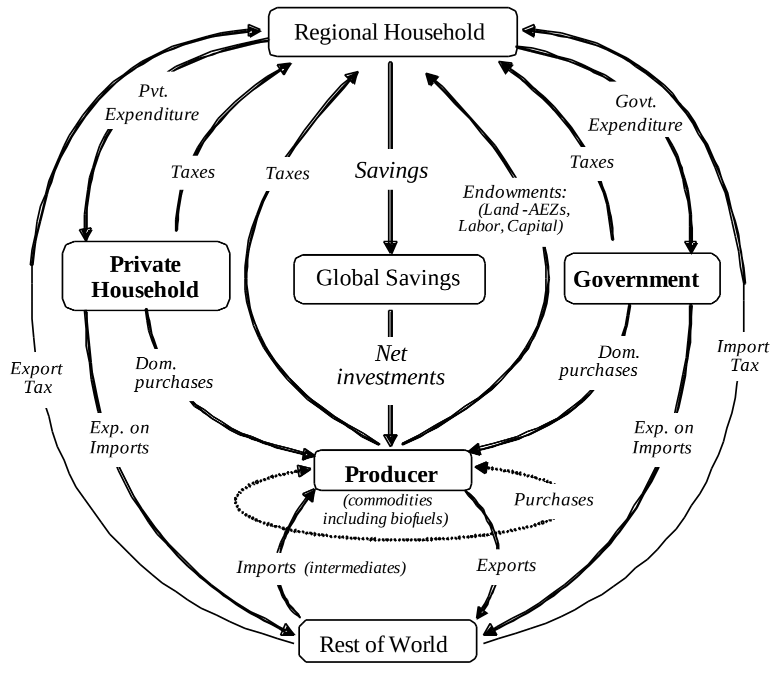

Supplementary Figure 22: Schematic of the Global Trade Analysis Project (GTAP) model. GTAP is a multi-commodity, multi-regional computable general equilibrium model documented in a book, published by Cambridge University Press[10] with detailed discussion on theory and derivation of the behavioral equations involved in the model. The standard GTAP model employs the simple, but robust, assumptions of constant returns to scale and perfect competition in all the markets with Walrasian adjustment to ensure a general equilibrium. As represented

in the figure below [11], the regional household (e.g., the EU) collects all the income in its region and spends it over three expenditure types – private household (consumer), government, and savings, as governed by a Cobb-Douglas utility function. A representative firm maximizes profits subject to a nested Constant Elasticity of Substitution (CES) production function which combines primary factors and intermediates inputs to produce a final good. Firms pay wages/rental rates to the regional household in return for the employment of land, labor, capital, and natural resources. Firms sell their output to other firms (intermediate inputs), to private households, government, and investment. Since this is a global model, firms also export the tradable commodities and import the intermediate inputs from other regions. These goods are assumed to be differentiated by region, following the Armington assumption, and so the model can track bilateral trade flows.

Agricultural land is imperfectly mobile across uses. Labor and capital markets are segmented, allowing for differential returns between the agriculture and non-agriculture sectors and immobile across countries. Government spending is modeled by using a Cobb-Douglas sub-utility function, which maintains constant expenditure shares across all budget items. The private household consumption is modeled with a non-homothetic Constant Difference of Elasticity (CDE) implicit expenditure function, which allows for differences in price and income elasticities across commodities. Taxes (and subsidies) go as net tax revenues (subsidy expenditures) to the regional household from private household, government, and the firms. The rest of the world gets revenues by exporting to private households, firms and government. In the GTAP model, this rest of world composite is actually made up of many other regions – with the same utility and production functions as for the regional household at the top of this figure.

In this paper, we employ the standard GTAP model closure which imposes equilibrium in all the markets, where firms earn zero-profits, the regional household is on its budget constraint, and global investment equals global savings. The global trade balance condition determines the world price of a given commodity.

GTAP Parameter	Description	Sector	Mean	Max	Min	Source
ETRAE	CET substitution parameter between sectors for sluggish primary factors—governs extensive margin of supply	Endowment Commodities				
		Land	-1.00	+/- 50%		Authors' Assumptions
ESUBVA	CES substitution parameter between primary factors in production – governs intensive margin of supply response	Produced Commodities				
		paddyrice	0.26	+/- 50%		Authors' Assumptions
		wheat	0.26			
		Crsgrns	0.26			
		Fruitveg	0.26			
		Oilsds	0.26			
		sugarcrps	0.26			
		Cotton	0.26			
		Othercrps	0.26			
		Livestock	0.26			
		PrFood	1.12			
		PrLstk	1.12			
		NRes	0.20			
		Mnfcing	1.26			
		Services	1.36			
		CGDS	1.00			
ESUBD	Armington CES substitution parameter for domestic / imported allocation—governs price responsiveness of trade/export demand	Tradeable Commodities				
		paddyrice	5.05	8.97	1.13	How Confident Can We Be in CGE-Based Assessments of Free Trade Agreements? By Hertel et al. GTAP Working Paper No. 26 2003[12]
		wheat	4.45	8.57	0.33	
		Crsgrns	1.30	2.38	0.22	
		Fruitveg	1.85	2.24	1.46	
		Oilsds	2.45	3.23	1.67	
		sugarcrps	2.70	4.66	0.74	
		Cotton	2.50	4.85	0.15	
		Othercrps	3.25	3.64	2.86	
		Livestock	2.06	2.74	1.37	
		PrFood	2.25	2.35	2.15	
		PrLstk	4.18	5.06	3.30	
		NRes	5.67	9.40	1.95	
		Mnfcing	3.54	3.73	3.34	
SUBPAR	CDE substitution parameter—governs price responsiveness of consumer demand	Tradeable Commodities				
			0.95-0.90	+/- 5%		Authors' Assumptions
			0.90-0.85	+/- 10%		
			0.85-0.80	+/- 20%		
			0.80-0.75	+/- 25%		
			0.75-0.70	+/- 35%		
			0.70-0.65	+/- 45%		
			<0.65	+/- 50%		

Supplementary Table 8: Parameters included in GTAP sensitivity analysis and distributions used. All distributions are treated as symmetric triangular distributions. Sets of GTAP parameters were varied jointly in the sensitivity analysis (i.e. all ESUBVA parameters were high or all were low). For computational reasons, a 16-region version of GTAP (with regions matching those in the FUND model) was used instead of the 140-region version used in the main text.

Supplementary References

1. Monfreda, C., Ramankutty, N. & Foley, J. A. Farming the planet: 2. Geographic distribution of crop areas, yields, physiological types, and net primary production in the year 2000. *Global Biogeochem. Cycles* **22,** n/a-n/a (2008).
2. Anthoff, D. & Tol, R. S. J. FUND v3.9 Scientific Documentation. (2014). Available at: http://www.fund-model.org/versions. (Accessed: 12th April 2016)
3. Challinor, A. J. *et al.* A meta-analysis of crop yield under climate change and adaptation. *Nat. Clim. Chang.* **4,** 287–291 (2014).
4. Wilcox, J. & Makowski, D. A meta-analysis of the predicted effects of climate change on wheat yields using simulation studies. *F. Crop. Res.* **156,** 180–190 (2014).
5. Kucharik, C. J. A multidecadal trend of earlier corn planting in the central USA. *Agron. J.* **98,** 1544–1550 (2006).
6. Seifert, C. A. & Lobell, D. B. Response of double cropping suitability to climate change in the United States. *Environ. Res. Lett.* **10,** 24002 (2015).
7. Menzel, A., Von Vopelius, J., Estrella, N., Schleip, C. & Dose, V. Farmers' Annual Activities are not Tracking the Speed of Climate Change. *Clim. Res.* **32,** 201–207 (2006).
8. Liu, B. *et al.* Similar estimates of temperature impacts on global wheat yield by three independent methods. *Nat. Clim. Chang.* **6,** 1130–1136 (2016).
9. Lobell, D. B. & Asseng, S. Comparing estimates of climate change impacts from process-based and statistical crop models. *Environ. Res. Lett.* **12,** 15001 (2017).
10. Hertel, T. W. *Global Trade Analysis: Models and Applications*. (Cambridge University Press, 1997).
11. Brockmeier, M. *A Graphical Exposition of the GTAP Model*. (2001).
12. Hertel, T., Hummels, D., Ivanic, M. & Keeney, R. *How Confident can we be in CGE-Based Assessments of Free Trade Agreements?* (2003).
